# Measurements of Atmospheric Proteinaceous Aerosol in the Arctic Using a Selective UHPLC/ESI-MS/MS Strategy

**DOI:** 10.1007/s13361-018-2009-8

**Published:** 2018-07-17

**Authors:** Farshid Mashayekhy Rad, Javier Zurita, Philippe Gilles, Laurens A. J. Rutgeerts, Ulrika Nilsson, Leopold L. Ilag, Caroline Leck

**Affiliations:** 1 Department of Environmental Science and Analytical Chemistry, Stockholm University, SE-106 91 Stockholm, Sweden; 2 Department of Meteorology, Stockholm University, SE-106 91 Stockholm, Sweden; 3 Department of Chemistry, KU Leuven, Celestijnenlaan 200F, Box 2404, 3001 Heverlee, Belgium

**Keywords:** Proteins, Amino acids, Arctic aerosols, LC/MS, Fixed-charge derivatization

## Abstract

In this article, an analytical methodology to investigate the proteinaceous content in atmospheric size-resolved aerosols collected at the Zeppelin observatory (79 °N, 12 °E) at Ny Ålesund, Svalbard, from September to December 2015, is proposed. Quantitative determination was performed after acidic hydrolysis using ultrahigh-performance liquid chromatography in reversed-phase mode coupled to electrospray ionization tandem mass spectrometry. Chromatographic separation, as well as specificity in the identification, was achieved by derivatization of the amino acids with *N*-butyl nicotinic acid *N*-hydroxysuccinimide ester prior to the analysis. The chromatographic run was performed within 11 min and instrumental levels of detection (LODs) were between 0.2 and 8.1 pg injected on the column, except for arginine which exhibited an LOD of 37 pg. Corresponding method LODs were between 0.01 and 1.9 fmol/m^3^, based on the average air sampling volume of 57 m^3^. The sum of free amino acids and hydrolyzed polyamino acids was shown to vary within 6–2914 and 0.02–1417 pmol/m^3^ for particles in sizes < 2 and 2–10 μm in equivalent aerodynamic diameter, respectively. Leucine, alanine, and valine were the most abundant among the amino acids in both aerosol size fractions. In an attempt to elucidate source areas of the collected aerosols, 5- to 10-day 3D backward trajectories reaching the sampling station were calculated. Overall, the method described here provides a first time estimate of the proteinaceous content, that is, the sum of free and polyamino acids, in size-resolved aerosols collected in the Arctic.

**Electronic supplementary material:**

The online version of this article (10.1007/s13361-018-2009-8) contains supplementary material, which is available to authorized users.

**Graphical Abstract:**
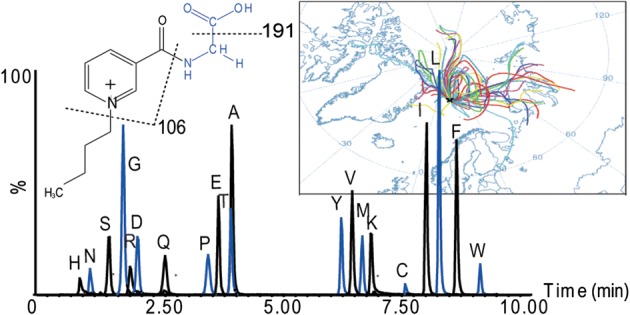
ᅟ

## Introduction

In the last two decades, a wide range of organic compounds has been measured in both polar and remote marine aerosols, clouds, fogs, and precipitation. In view of this and Saxena’s discovery of proteinaceous material in Antarctic cloud water samples [1], and Orellanas et al.’s and Verdugo’s later demonstrations of marine biogenic polymer or marine gels in the high Arctic cloud droplets [2, 3], proteins together with the polysaccharides are likely present within the thin surface film at the water-air interface, referred to as the surface ocean microlayer (SML). This was also suggested by Gershey, who found that production of aerosols by bubbling in seawater discriminated against the more soluble low molecular weight compounds in favor of the more surface active high molecular weight compounds [4]. Furthermore, Gao et al. examined the foam produced by bubbling seawater collected in the central Arctic Ocean, finding that it contained highly surface-active marine gels [5]. Also, a large number of gel particles were detected within the SML at the water-air interface between high Arctic ice floes, as reported by Orellana et al. and Bigg et al. [2, 6]. The findings by Gao et al., Bigg et al., and Orellana et al. are consistent with those of Aller et al. [7], Bigg and Leck [8], Kuznetsova et al. [9], Leck and Bigg [10], Leck et al. [11], Matsumoto and Uematsu [12], and Zhou et al. [13], which showed most of the sea spray injected into the atmosphere by bubble bursting from the SML contained both polysaccharides and proteins.

The release of amino acids from proteins can occur by enzymatic hydrolysis in an aqueous medium with bacteria providing the enzymes [14]. In Bigg and Leck’s publication [8], it was suggested that marine gels, as well as associated particulate matter such as bacteria, phytoplanktons, and its detritus, can be carried selectively to the SML by rising bubbles. Following the burst of the bubbles at the water-air interface, the drop fragments would not only carry the polymer saccharides, but also any protein, amino acid, or bacteria with its associated enzymes attached. In an article by Leck and Bigg [10], an example is given. According to Edna Graneli (personal communication 2001), such enzymes could survive up to 3 days after injection into the atmosphere.

The assumption that proteins are subjected to degradation in the atmosphere has been supported by the fact that, considering enrichment factors, a greater presence of free amino acids (FAAs) has been observed in marine aerosol and precipitation relative to seawater. Typical levels of the dissolved free amino acids measured in marine rain by Mopper and Zika showed enrichments by two orders of magnitude over typical seawater values [15]. Spitzy reported amino acid data from size-fractionated aerosol and rain collected over the northern Indian Ocean [16]. The author found a several-fold enrichment of amino acid-derived nitrogen in the airborne aerosol sub-micrometer size fraction over the coarse fraction. The levels measured in rain samples [16] were generally in accordance with those observed by Mopper and Zika [15]. Furthermore, the latter authors found that L-methionine in rainwater samples was usually detected almost exclusively in its oxidized form. This led to the suggestion by Leck and Bigg [17] that the aqueous oxidation of L-methionine, mainly through OH radicals [18], could possibly be involved in the nucleation of new particles in the atmosphere via condensable vapor.

While FAAs from the remote marine atmosphere have been reported in both particulate and condensed phases (rainwater), polyamino acids (PAAs) which indicate the existence of peptides and proteins have been less investigated and their levels in the atmosphere are still a subject of discussion [9, 19]. In particular, there is essentially no information regarding PAA levels in Arctic areas [20]. One likely reason for that is the difficulty to determine PAAs at trace levels due to the variety of types, as well as the diversity of sources, such as parts of bacteria, sea ice algae, phytoplanktons, and their secretions, thus being present in many forms in terms of amino acid sequence.

Different techniques, such as paper chromatography [21], capillary electrophoresis [22], gas chromatography (GC) with flame ionization detection [23], and high-performance liquid chromatography (HPLC) with UV or fluorescence detection [24, 25], as well as GC/mass spectrometry (MS) [26] and HPLC/MS methods [27], have been commonly employed for amino acid determination. In the last years, the use of high-resolution mass spectrometry and tandem mass spectrometry (MS/MS) has contributed with higher selectivity. Samy et al. employed ion-pairing HPLC coupled to MS/MS [28], including time-of-flight, to separate and determine FAAs in airborne aerosol sub-micrometer size fraction from the southeast of the USA. Alternatively, hydrophilic interaction chromatography (HILIC) has been used together with electrospray ionization (ESI) triple quadrupole MS for detection of FAAs in the Arctic [29]. Chiral chromatography has also been used by Barbaro et al. to determine the amino acid composition in size-segregated atmospheric aerosols (< 0.49 μm and up to 10 μm) over Antarctica [30]. An excellent review by Matos et al. has been published recently regarding challenges in the measurements of FAAs and proteinaceous compounds in atmospheric aerosols [20]. These mainly revolve around the variety of approaches that have been used that preclude direct comparisons of results. A common challenge mentioned was the detection of all amino acids given instabilities associated with certain aspects of the workflow, such as acidic hydrolysis.

This study focuses on the development of a fast and sensitive reversed-phase HPLC/ESI-MS/MS methodology, including derivatization, to enable measurement and profiling of total amino acids (TAAs), i.e., both FAAs and PAAs after acidic hydrolysis, in atmospheric size-resolved aerosol particles collected at Ny Ålesund, Svalbard, during autumn and winter 2015. Hydrolysis was applied since the diversity of PAAs is expected to be very high together with low levels, making the measurement of individual PAAs a challenge. By measuring TAAs, i.e., the sum of FAAs and hydrolyzed PAAs, important information is provided on the total amount of proteinaceous material present in the aerosols in the high Arctic.

Another novelty presented here, with respect to the measurement of amino acids in atmospheric aerosols, is the use of the derivatization reagent *N*-butyl nicotinic acid *N*-hydroxysuccinimide ester (C_4_-NA-NHS). In addition to the benefit of adding a permanent charge, the use of this tag increases the specificity in the amino acid identification. A signal enhancement when compared to other hydrophobic tags and non-derivatized amino acids has been suggested [31].

## Material and Methods

### Chemicals

Standard amino acids including L-alanine (Ala), L-arginine (Arg), L-asparagine (Asn), L-aspartic acid (Asp), L-cysteine (Cys), L-glutamic acid (Glu), L-glutamine (Gln), glycine (Gly), L-histidine (His), L-isoleucine (Ile), L-leucine (Leu), L-lysine (Lys), L-methionine (Met), L-phenylalanine (Phe), L-proline (Pro), L-serine (Ser), L-threonine (Thr), L-tryptophan (Trp), L-tyrosine (Tyr), L-valine (Val), and surrogate internal standards (IS) including the deuterated amino acids Phe-D_5_, Gln-D_5_, Glu-D_5_, and Leu-D_3_, as well as trifluoroacetic acid (TFA) and human serum albumin (HSA) both of > 97% purity, were all purchased from Sigma-Aldrich (St. Louis, MO, USA). The purity was higher than 99% for all amino acids. Ultrapure water (resistivity > 18 MΩ cm) was obtained from a Milli-Q plus system from Millipore (Bedford, MO, USA). Acetonitrile of LC/MS grade was purchased from VWR (Radnor, PA, USA). Acetic acid of 99.9% was purchased from Scharlau (Barcelona, Spain) and hydrochloric acid (HCl) of 36% purity was purchased from Sigma-Aldrich. Ammonium borate (100 mM) buffer at pH 9 was obtained from Waters (Milford, MA, USA). *N*-Butyl nicotinic acid *N*-hydroxysuccinimide ester (C_4_-NA-NHS) was synthesized in-house at the University of Leuven, Department of Chemistry (Heverlee, Belgium), following previously published work by Yang et al. [31].

### Sampling Location and Period

Size-resolved aerosol samples were collected at the Zeppelin observatory at Ny Ålesund, Svalbard (79° N, 12° E, 474 m above sea level, a.s.l.), from September to December in 2015. The sampling was part of a multidisciplinary program that studied atmospheric chemistry and meteorology in the Arctic region to raise our knowledge regarding remote marine aerosols originating from within the marginal ice zone or in the open waters south thereof. The program was coordinated by the Norwegian Institute for Air Research (NILU).

### Sampling of Airborne Particles

A two-stage stacked filter unit (SFU) sampler [32] that operated at a flow rate of 17 standard liters per min (slpm) was employed for the collection of airborne particles. The sampling times for the samplers varied between 2 and 3 days, resulting in an average air sampling volume of 57 m^3^. In total, 40 size-resolved samples were collected. Blank samples were obtained by having no air drawn through the SFU during the length of the sampling period.

The SFU contained a coarse (8 μm pore size) filter and a fine (0.4 μm pore size) Whatman® Nuclepore™ polycarbonate filter, respectively. The filters were bought from Merck Millipore Company (Billerica, MA, USA). The coarse filter had a 50% collection efficiency at 2-μm equivalent aerodynamic diameter, EAD [33], and thus collected particles within the size range 2–10 μm EAD, whereas the fine filter collected particles < 2 μm EAD. The SFU was operated downstream of a cyclone inlet at ambient conditions, which removed particles (and cloud elements) above 10 μm EAD. In order to minimize contamination of the filter substrates (ambient and blank), the samplers were changed in a glove box (free from particles, sulfur dioxide, and ammonia) both before and after sampling. Further, to avoid high chemical blank values, all filter substrates were cleaned by ethanol and ultrapure water and then dried before use. All filter substrates were stored at – 80 °C prior to the determinations of TAAs, including both FAAs and PAAs.

### Standard Solutions and Derivatization Procedure

Individual stock solutions of amino acids, as well as of the deuterated IS Phe-D_5_, Gln-D_5_, Glu-D_5_, and Leu-D_3_, were prepared at a concentration of 50 mM in 20 mM HCl (aq). The four IS were mixed and diluted in ultrapure water to a final concentration of 250 nM. Working standard solutions including a mixture of 20 amino acids at six concentration levels ranging from 0.005 to 10 μM were obtained by dilutions in ultrapure water. A volume of 15 μL was taken out from each concentration level followed by addition of 15 μL of the IS solution. Derivatization according to Yang et al. [31] was then performed by adding 60 μL of borate buffer (100 mM, pH 9) and 20 μL of C_4_-NA-NHS solution (40 mg/mL in acetonitrile). The solution was vortexed for 10 s at room temperature and was then immediately transferred to speed-vac for evaporation, after which it was reconstituted in 15 μL of mobile phase A (see below, section “Sample Preparation”) prior to UHPLC/ESI-MS/MS analysis.

### Sample Preparation

Prior to the determination of TAAs, each filter substrate with the collected aerosols was ultrasonically solvent-extracted twice for 15 min, each time in 10 mL of ultrapure water. The combined extracts were then transferred to a Pyrex glass flask. All glassware used had previously been heated for 2 h at 550 °C to remove any possible source of contamination. The extract was then vacuum-dried by a rotary evaporator (RII, BUCHI, Switzerland) at 40 °C and reconstituted in 250 μL of acetonitrile/water (80/20 *v*/*v*). After extraction, an aliquot of 15 μL from this solution was taken, dried, and reconstituted in 15 μL of ultrapure water and a 15-μL portion of the IS mixture (see section “Standard Solutions and Derivatization Procedure”) was added. For hydrolysis, a 15-μL volume of TFA/HCl (1:2 *v*/*v*) was added, after which the samples were incubated at 165 °C for 35 min, following a procedure by Tsugita and Schefler [34]. After hydrolysis, excess acid was removed using a vacuum centrifuge, followed by derivatization as described for the standard solutions (section “Standard Solutions and Derivatization Procedure”). Filter blanks were treated in the same way as the samples, and subsequently, blank values were calculated and subtracted for each amino acid prior to quantification.

HSA was hydrolyzed and derivatized under the same conditions as for the samples and was used as a model for PAAs in order to test the efficiency of the hydrolysis. A comparison between the theoretical and experimental amino acid composition and experimental after hydrolysis is shown in Electronic Supplementary Material, Table S-1.

### Preparation of Solutions to Check for Matrix Effects

In order to check for any matrix effects on the ES ionization from the sample clean-up, matrix-adapted standard solutions were prepared. Filter blanks were used as matrix and were spiked with standard solutions at the same levels as described in section “Standard Solutions and Derivatization Procedure.” The spiked samples were treated in the same way as the samples and subjected to hydrolysis as described in section “Sample Preparation” including derivatization, except that no surrogate deuterated IS was added before or after hydrolysis.

### Ultrahigh-Performance Liquid Chromatography

Ultrahigh-performance liquid chromatography (UHPLC) separation was accomplished on an Acquity UPLC system (Waters, MA, USA) using an Acquity UPLC BEH column C18 (Waters, 50 × 2.1 mm, 1.7 μm particle size). The mobile phase compositions were (a) ultrapure water with 0.3% acetic acid (*v*/*v*) and (b) acetonitrile with 0.3% acetic acid (*v*/*v*). The flow rate was 800 μL/min. A gradient program was applied as follows: 0–2.50 min 100% A; 2.51–4.50 min 0.5–3% B; 4.51–7.50 min 5–9% B; 7.51–9.49 min 12–15.5% B. Then conditioning of the column was performed from 9.5 to 10 min with 95% B and equilibration with initial conditions from 10.01 to 11 min. The total run time was 11 min.

### Tandem Mass Spectrometry

C_4_-NA-NHS derivatives of amino acids and internal standards were determined using a triple-quadrupole mass spectrometer (Xevo TQ-S Micro, Waters, MA, USA) with positive ESI. The optimized conditions, monitored transitions, and time descriptors for each amino acid derivative are listed in Table S-2 in Electronic Supplementary Material. Quantification was carried out in multiple reaction monitoring (MRM) mode. Data were obtained and processed using MassLynx 4.1 MS software package from Waters.

### Method Evaluation

The linearity was evaluated by fitting linear regressions to the calibration curves and the square of the correlation coefficients (*R*^2^) was determined. The calibration curve showed *R*^2^ value greater than 0.99. The instrumental limits of detection (LODs) and limits of quantification (LOQs) for individual analytes were estimated experimentally using serially diluted standard solutions until a signal-to-noise ratio (S/N) of 3 and 10, respectively. The method LODs were determined as femtomole per cubic meter of sampled air, based on the average air sample volume of 57 m^3^, with the same criteria for S/N. Method lack of precision as % RSD values and accuracy were measured for triplicate quality control samples (QC) at 1.5, 6, and 8 μM concentration levels. All the method parameters evaluated are listed in Table S-3 in Electronic Supplementary Material.

### Trajectory Analyses

3D back trajectories have been calculated for the receptor site on Mt. Zeppelin near Ny Ålesund, Svalbard, at 500 m a.s.l. The trajectories were computed backwards for 5 to 10 days at 6-h intervals, using the HYSPLIT2 model [35] with GDAS data (Global Data Assimilation System). More information about the GDAS data set can be found from the Air Resources Laboratory (ARL), National Oceanic and Atmospheric Administration NOAA web server (http://ready.arl.noaa.gov/).

## Evaluation of the Analytical Methodology Proposed to Investigate Proteinaceous Matter in Atmospheric Aerosols

### Mass Spectrometry of C_4_-NA-NHS Tagged Amino Acids

One common problem with analyzing environmental samples using ESI is ion suppression from matrix compounds, such as salt and soot. Additionally, when using reversed-phase HPLC for amino acids, this problem is often enhanced for polar species eluting close to the void volume together with weakly retained interferences. The polar behavior also often decreases the ESI signals per se, especially under the highly aqueous conditions often applied in reversed-phase HPLC. Derivatization with the commercial tag aminoquinolyl-*N*-hydroxysuccinimidyl carbamate (AQC) has become a common strategy to improve both reversed-phase retention and ESI responses of amino acids but these offer no specificity.

**Figure 1:**
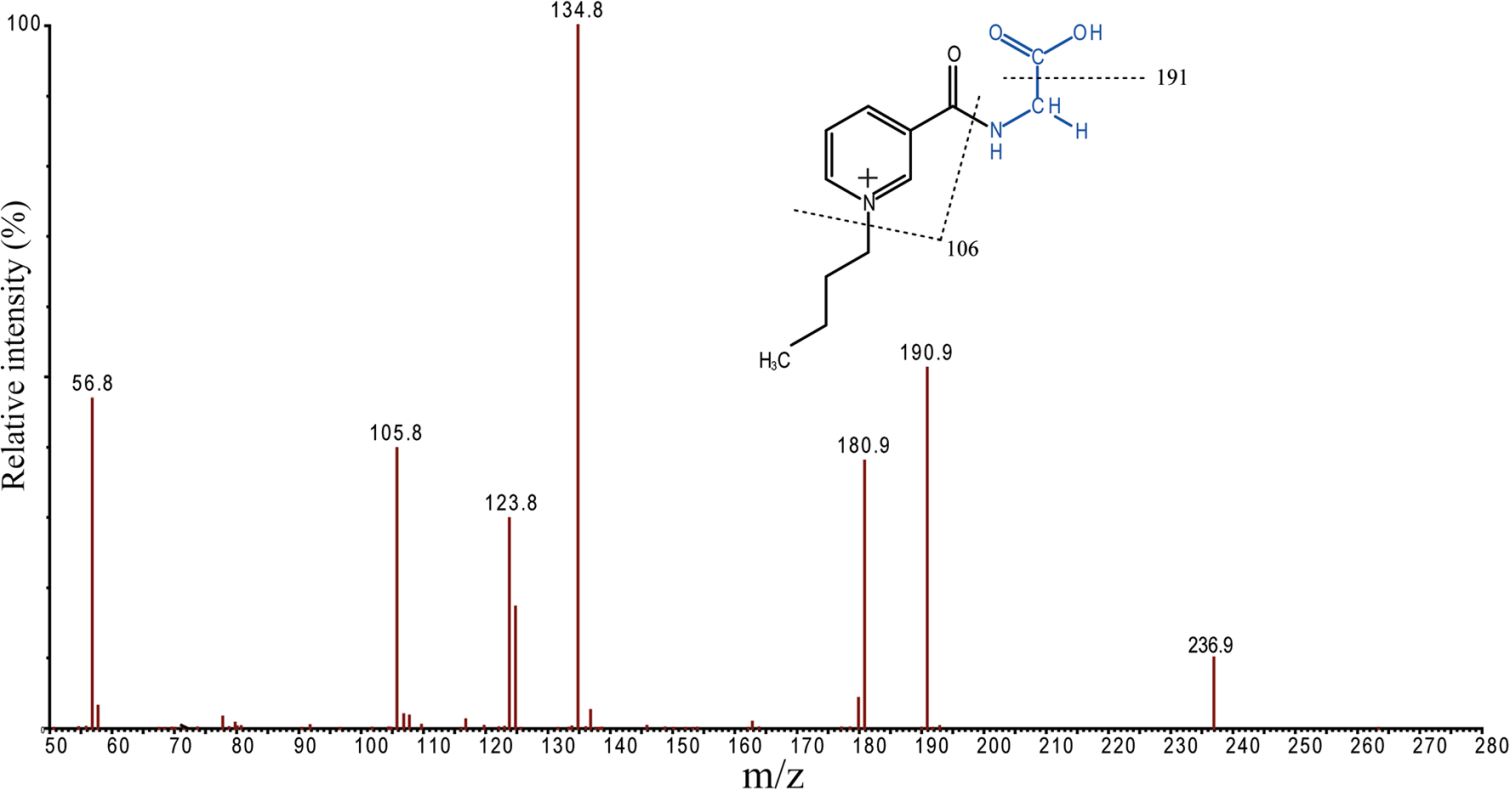
An MS/MS product ion scan spectrum of the parent ion from C_4_-NA-NHS tagged Gly

**Figure 2:**
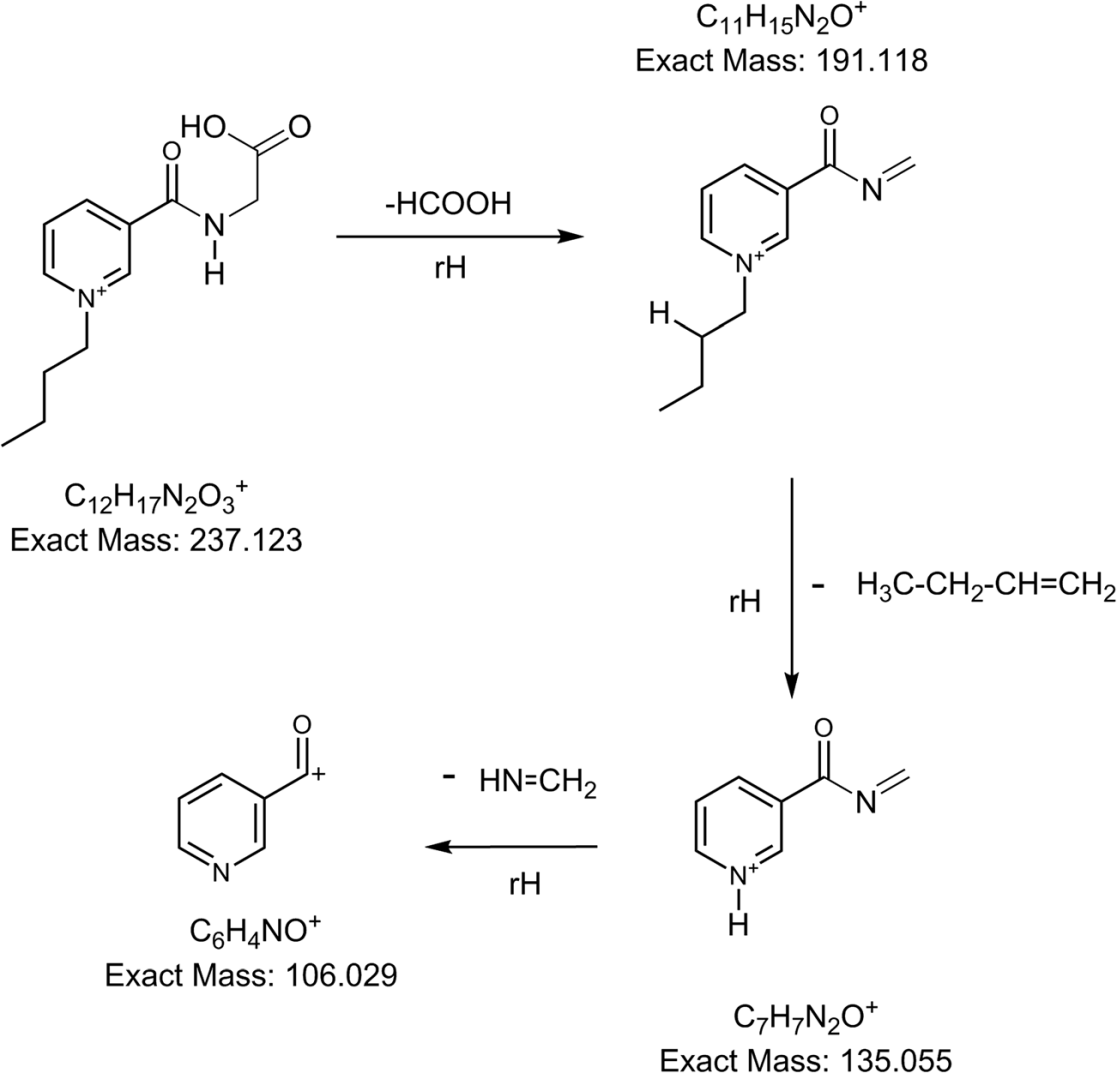
Suggested mechanisms for the fragmentation of the parent ion from derivatized Gly in ESI-MS/MS

To achieve the highest possible selectivity for the C_4_-NA-NHS tagged amino acids in the present work, two transitions from each parent ion [M]+ in MRM mode were used (see Table 1). The transition from precursors to *m*/*z* 106 was common for all derivatized analytes, corresponding to formation of [pyridine-CO]+ from the C_4_-NA-NHS tag moiety. Contrary to the widely used and commercially available AQC tag, our derivatives also yield ion transitions that are specific for each amino acid [36]. AQC derivatives, on the other hand, exhibit only one fragment of sufficient signal strength (at *m*/*z* 171), common for all amino acids [37, 38]. The specific transitions we obtained are highly valuable since they strengthen the identification. As an example, the product ion spectrum of tagged Gly is shown in Figure 1. In Figure 2, the main fragmentation pathways for this derivative are shown, with the ion transition from [M]+ to *m*/*z* 135 being specific for Gly. Isobaric Ile and Leu derivatives are the only indistinguishable analytes under the conditions used. All MRM transitions used are given in Table S-2 in Electronic Supplementary Material.

**Table 1:** The 25th, 50th (Median), and 75th Percentile of TAAs and the Individual Amino Acids Determined in the SFU Samples Collected at the Zeppelin Observatory in 2015

	TAAs	Amino acids (pmol/m^3^)															
Size range	pmol/m^3^	Ala	Arg	Asp	Cys^d^	Glu	Gly	His	Ile	Leu	Lys	Met	Phe	Pro	Ser	Thr	Val
Fine mode^a^																	
25th percentile	35.6	4.6	0.0	^b^n.d	^b^n.d	2.4	1.6	^b^n.d	2.4	4.3	1.3	0.1	2.1	0.9	^b^n.d	2.1	3.6
50th percentile	105	11.2	2.0	^b^n.d	^b^n.d	7.3	4.5	^b^n.d	5.9	12.2	5.3	1.1	7.7	2.9	^b^n.d	4.6	9.6
75th percentile	298	33.4	6.4	^b^n.d	^b^n.d	19.7	64.2	^b^n.d	10.5	31.2	14.1	3.0	13.7	14.7	^b^n.d	15.8	22.9
Max	2914	402	216	0.001	19	172	650	^b^n.d	178	392	163	20	134	275	205	263	264
Min	5.6	0.73	^b^n.d	9.8	^b^n.d	^b^n.d	^b^n.d	^b^n.d	^b^n.d	0.37	^b^n.d	^b^n.d	^b^n.d	^b^n.d	^b^n.d	0.24	0.17
Coarse mode^a^																	
25th percentile	26.6	3.6	0.1	^b^n.d	^b^n.d	2.0	0.6	^b^n.d	1.5	4.4	1.7	0.1	2.1	0.6	^b^n.d	1.4	2.8
50th percentile	46.9	7.1	1.7	^b^n.d	^b^n.d	4.5	4.2	^b^n.d	3.0	6.6	3.6	0.5	3.6	1.6	^b^n.d	2.7	5.3
75th percentile	219.3	16.6	6.5	0.1	^b^n.d	9.1	58.3	^b^n.d	6.6	16.7	7.6	2.2	8.1	7.9	^b^n.d	6.1	9.6
Max	1417	120	82	28	6.5	135	321	^b^n.d	98	228	102	22	87	65	53	80	106
Min	0.018	0.0001	0.008	0.001	^b^n.d	^b^n.d	0.001	^b^n.d	^b^n.d	^b^n.d	0.0003	0.0001	0.0001	0.001	0.0001	0.0003	^b^n.d
FF^c^																	
25th percentile	0.27	0.35	0.14	^b^n.d	^b^n.d	0.30	0.24	^b^n.d	0.27	0.29	0.25	0.08	0.24	0.43	^b^n.d	0.47	0.32
50th percentile	0.64	0.59	0.50	^b^n.d	^b^n.d	0.59	0.66	^b^n.d	0.65	0.65	0.48	0.50	0.59	0.61	^b^n.d	0.62	0.60
75th percentile	0.80	0.79	0.78	^b^n.d	^b^n.d	0.86	0.90	^b^n.d	0.86	0.83	0.81	0.97	0.79	0.87	^b^n.d	0.82	0.80

^a^Particles in the fine mode size range (< 2 μm EAD) and coarse mode size range (2 < EAD < 10 μm)

^b^n.d = the value is below method LOD

^c^Fine molar fraction (FF) in percentage is calculated by median of ((Dp < 2 μm) / (Dp < 2 μm) + (2 < Dp < 10 μm)) * 100

^d^Cys was measured as cysteine

**Figure 3:**
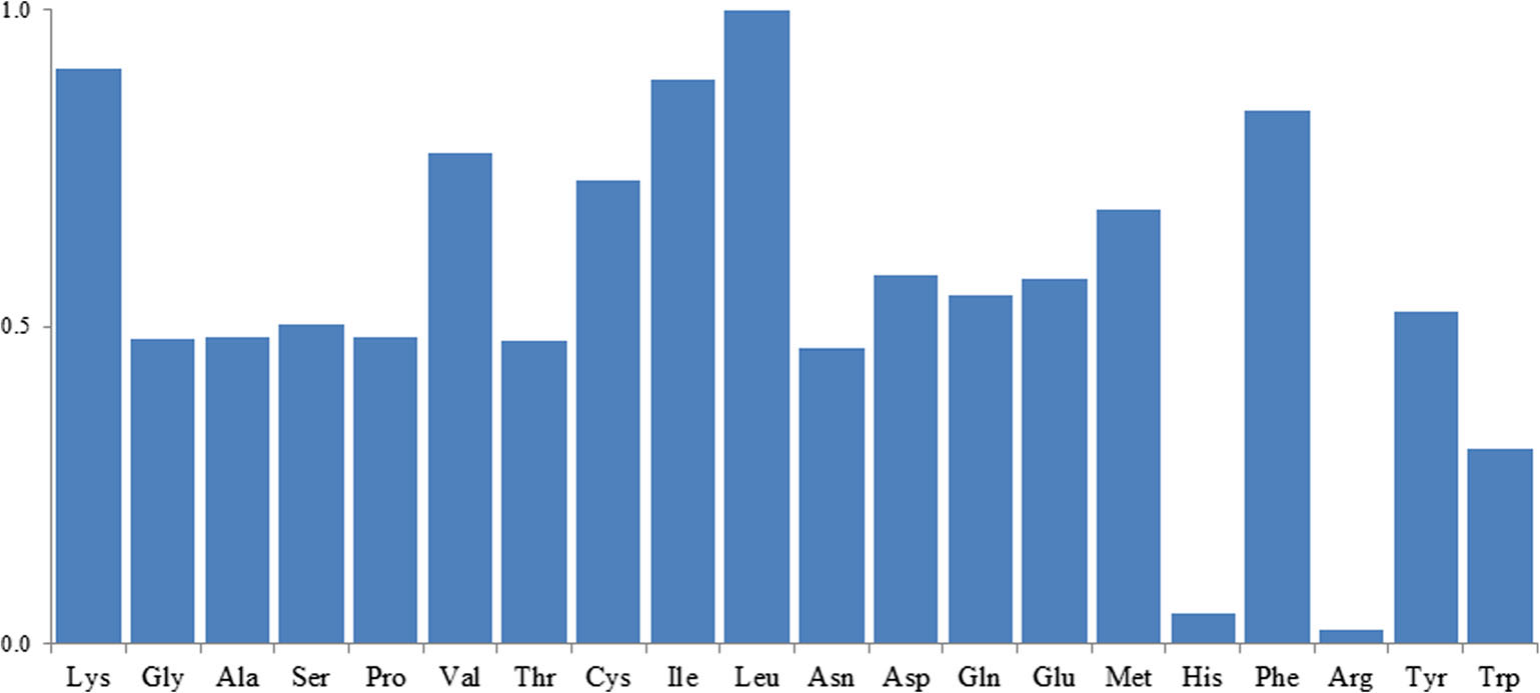
The relative signal response at LC/ESI-MS/MS from the different amino acid standard derivatives. Each response is normalized to the signal of Leu

The obtained instrumental LOD values in the present work (Table S-3 in Electronic Supplementary Material) were shown to be comparable (0.2–37 pg, corresponding to 0.004–7.5 nM for sample volumes of 15 μL) to those reported for AQC-tagged amino acids in the analysis of *Arabidopsis* leaf tissue [38]. The MS response factors for the derivatives were shown to vary depending on the amino acid side chain, as shown in Figure 3, where all responses are normalized to the Leu derivative. Tagged Leu showed the highest response, while the more hydrophilic Arg derivative showed the lowest. This can be explained by a lower tendency to be ionized and/or be desorbed from the droplet surface in ESI. The latter can be particularly true for amino acids with basic side chains such as Arg and His which have the capability of being protonated, thus adding a second charge and decreasing the droplet surface activity.

### Optimization of LC Conditions

**Figure 4:**
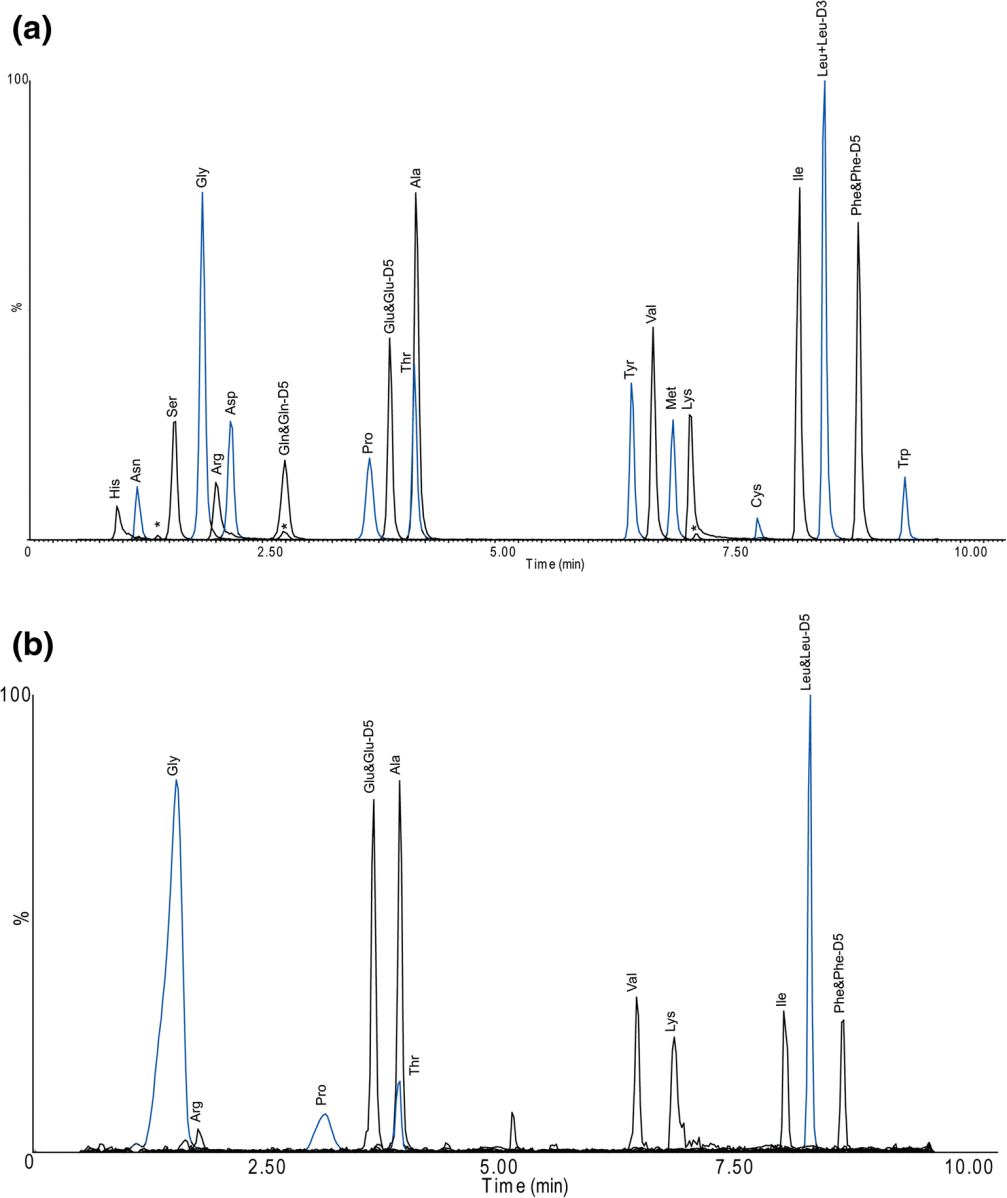
(**a**) Total ion current chromatogram in MRM mode of a standard solution containing tagged amino acids. Two transitions were monitored for each analyte (see Table S-2). The LC conditions are given in experimental, section “Ultrahigh-Performance Liquid Chromatography.” (**b**) Total ion current chromatogram in MRM mode of a fine mode aerosol (< 2 μm) sample collected during Sep. 23–25 in 2015. The same LC conditions as in (**a**) were used

Chromatographic resolution of amino acids on the C18 column is challenging due to the wide range of both log P and p*K*a. However, full separation could be achieved for almost all tagged amino acids, except for derivatives of Thr and Ala, as shown in the total ion chromatogram in Figure 4a. The latter two could be separated by specific *m*/*z* transitions.

A major advantage of the presented method is the fast separation of 20 amino acids, with the entire chromatographic run performed within 11 min by using the binary solvent gradient system described in section “Ultrahigh-Performance Liquid Chromatography.”

### Method Linearity, Accuracy and Precision, and Levels of Detection and Quantification

Use of isotope-labeled reference compounds is generally favorable for quantification in order to compensate for compound-dependent matrix effects; however, this can be expensive when using one for each amino acid. Therefore, in this study, we tested the variability of the matrix effect in ESI among the different tagged amino acids, as shown in Table S-3 (Electronic Supplementary Material). In this case, the matrix was defined as composed of any interferences from blank filters and the analytical chain, such as acids from the hydrolysis, see “Material and Methods” section “Sample Preparation.” The signal in matrix-adapted standard solutions relative to the matrix-free standard solutions (the latter described in section “Standard Solutions and Derivatization Procedure” and had not been subjected to the hydrolysis conditions, but only to derivatization) varied between 62 and 97% with a mean value of 79% and an RSD of 12%. This variation was considered low enough to motivate the use of only four deuterated IS, although with retention times distributed over the entire chromatogram (see Figure 4a).

Linearity, LOD, accuracy, and precision of the developed method are also summarized in Table S-3 in Electronic Supplementary Material. For most of the amino acid derivatives, the LODs were between 0.2 and 8.1 pg injected amount, except for the tagged Arg. For the latter, its hydrophilicity is most likely the reason for the much higher LOD value (37 pg), as discussed in section “Mass Spectrometry of C_4_-NA-NHS Tagged Amino Acids.” The lowest reported LOD values so far, to our knowledge, have been reported by Barbaro et al. [30]. However, the fast separation achieved (only 11 min) in the present study, with common reversed-phase chromatography, is a viable alternative to their method based on chiral chromatography. Method LOD for our 40 SFU samples (in all 80 filter substrates) varied between 0.01 and 1.9 fmol/m^3^, based on the average air sampling volume of 57 m^3^.

Figure 4b shows a representative chromatogram for one of the samples. It shows a full separation of the amino acid derivatives, and for several of them, a high signal-to-noise ratio was achieved. Occasionally, in some of the chromatograms, the early eluting derivatives exhibited severe peak broadening. This occurred when the corresponding filter substrates contained unusually high levels of sea salt co-deposited on the substrates during collection.

### Evaluation of the Hydrolysis Experiment

For all types of hydrolysis of proteins, both acidic and enzymatic, it is reasonable to expect variable degrees of amino acid losses, for instance, due to degradation or inefficient cleavage of the peptide bonds. However, substantial difference in recoveries can unfortunately lead to an incorrect measure of the relative abundances of individual amino acids constituting peptides/proteins. The recent review by Matos et al. [20] showed that even the most commonly used method for hydrolysis (i.e., using 12 M HCl at 110 °C for 24 h) will result in losses of some amino acids. For that reason, this commonly used method was compared to a faster method for hydrolysis (HCl/TFA 1:1 *v*/*v* at 165 °C for 25 min) in this study. In the comparison, HSA was used as a model protein. The results showed very similar recoveries, except for Glu + Gln (combined, measured as Glu), Gly, Ala, and Asp + Asn (combined, measured as Asp), for which the faster hydrolysis approach gave significantly higher recoveries. Due to these results, the faster method was applied in the determinations of the collected atmospheric aerosol samples.

The found amino acid composition of HSA was compared with the theoretical relative amino acid abundances (Table S-1 in Electronic Supplementary Material). The results showed that Lys, Ala, Val, Cys (measured as cysteine), Ile, Leu, Glu + Gln, Met, His, Arg, and Ser were within 70–113% of the theoretical levels. The corresponding recoveries for other amino acids were as follows: for Phe 125%, for Pro 127%, and for Gly, Thr, and Asp + Asn 51–63%. Deviations observed for both Tyr and Trp were substantial (Table S-1), but the reason for this was not investigated and Tyr and Trp were not further considered in the estimates of the proteinaceous content of the aerosol samples collected in this study. Furthermore, the recoveries of Asn and Gln could not be measured per se since both were converted to Asp and Glu in the hydrolysis, respectively. As such, an unknown portion of the determined levels of Asp and Glu could have been present as Asn and Gln, respectively, in the aerosol collected.

## Proteinaceous Matter in the Arctic Atmospheric Aerosol

### Basic Statistics of TAAs Determined in Aerosol Fine and Coarse Modes

Table 1 gives basic descriptive statistics of the individual amino acids determined during the sampling period from September to December in 2015. The data are shown separately for the two SFU stages, which represent the fine (< 2 μm EAD) and coarse (2–10 μm EAD) modes, respectively, of the sampled aerosols. The 25th, 50th (median), and 75th percentile concentrations of both TAAs and of each individual amino acid are shown in Table 1, as well as the full concentration range of the same amino acids. Table 1 also tabulates the median molar ratio in % of the aerosol fine mode mass concentration to the mass concentration of TAAs summed over both the fine and coarse mode.

As seen in Table 1, the TAAs ranged from 5.6 to 2914 pmol/m^3^, with a median of 105 pmol/m^3^, for aerosol particles < 2 μm EAD; and from 0.02 to 1417 pmol/m^3^, with median of 47 pmol/m^3^, for aerosol particles between 2 and 10 μm EAD. Generally, the predominant amino acids in the fine mode were Leu, Ala, and Val. They accounted for slightly more than 10% each, at the 50th percentile, of the TAA content. For the coarse mode, the same amino acids were observed to be most prominent and accounted for between 11 and 15% at the 50th percentile of the TAA content. The amino acids Leu and Ala were the most abundant in both aerosol size fractions. The least detected amino acids were Met, Arg, and Pro, which contributed 1–3 and 1–4% in the fine and coarse modes, respectively, at the 50th percentile level. Notably below their respective method LODs were the amino acids Asp, Cys, His, and Ser. In the majority of the samples, the TAA levels in the fine mode were slightly elevated (ca. 60% at the 50th percentile level) compared to the coarse mode. Arg and Lys are two examples of amino acids being evenly distributed between the fine and coarse modes. In the case of Met, as much as 97% of the mass at the 75th percentile level was associated with the smallest particles collected. These results except for Met seem slightly surprising in view of the findings by Spitzy [16] that found a several-fold enrichment of amino acid nitrogen in the fine aerosol size fraction over the coarse fraction.

The measured TAA concentration is significantly higher (around three orders of magnitude) than FAA concentrations reported in Arctic aerosols [29], indicating that amino acids were present predominantly as PAAs. In this regard, our results are not directly comparable to the study by Scalabrin et al. in 2012 [29], or similar studies done elsewhere [12, 18], which included protein quantification but only in the intact form. In addition, sampling was performed across different years and seasons, e.g., spring through early autumn and autumn through winter in this study. Nonetheless, similar to the results obtained in the study by Scalabrin et al., one of the most abundant amino acids detected in this study was Gly (cf. Table 1 at the 75th percentile). The finding by Mopper and Zika [15] that Met was usually detected almost exclusively in its oxidized form in the atmosphere is consistent with the obtained low levels of Met described here and in the abovementioned study by Scalabrin et al.

### Atmospheric TAAs and Possible Sources and Transformations

A variety of sources and removal mechanisms may contribute to the relative abundance of amino acids in the atmospheric aerosol collected at the Zeppelin observatory during this study:

1. Long-range transported (> 5 days) biomass burning or pollution plumes of aerosol fine mode from high-temperature sources, with large plume lifts, on islands and/or land areas. As such, the pollution plumes can be injected directly into the free troposphere (FT) and are usually advected for days in the FT well above the top (ca. 1000 m a.s.l.) of the boundary layer (BL). Prior to arrival at the observatory (474 m a.s.l.), the plumes show a subsiding pathway [39].
2. Transport of marine biogenic particles within the BL from the marginal ice zone and open waters south thereof [2, 40–42]. This was suggested to involve fresh sea spray emissions at the sea-air interface of both primary fine mode aerosols (mainly organic in nature, e.g., polymer gels) derived from fragments of the bubble membrane (film) that are thrown into the air when bubbles burst (“film drops”), and the larger coarse mode particles (mostly sea salt but could contain an appreciable organic component, such as from bacteria and their attached enzymes) derived from drops of water that are detached from an upward moving jet of water that follows the bubble burst (“jet drops”).
3. The removal mechanisms of the particles from the atmosphere also set the limits on their atmospheric residence times, which in turn will indicate how far or close from the observatory their sources were located. Fine mode aerosols are primarily removed from the atmosphere through wet deposition and coarse mode aerosols through sedimentation. The estimated atmospheric aerosol residence time of the fine mode is in the range of 5–10 days, while it is less than 1 day for the coarse mode [43].
4. The synoptic-scale systems advecting heat, moisture, and aerosols from the above variety of sources for a variable length of time before arrival to the Zeppelin observatory will also affect the chemical and physical transformations of the airborne proteinaceous matter and hence the contributions from PAAs and FAAs to the relative abundance of amino acids of the observed sub- and super-micrometer aerosol collected. Consequently, the determined relative abundance of amino acids (i.e., fingerprint) ideally could be a useful indicator as to the origin or age of the aerosols collected during this study. For example, amino acids from distant sources (*referred to as Group A*) include Ala, Asp, Glu, and Gly. The three amino acids Gly, Glu, and Ala are relatively unreactive amino acids in the atmosphere where the first two have the lowest photochemical reactivity with half-life of 85 and 19 days, respectively [44], and Ala has been shown to rarely react with the hydroxyl radical and other radicals in the atmosphere [18]. It has been suggested therefore that Gly could be considered as a long-lived amino acid also in aerosols [12] and thus be used as an indicator for long-distance transport [44]. As such, the grouping of Gly, Glu, and Ala is suggested to be derived from thermal alteration derivatives from proteins [45] associated with residential heating in being most frequent during autumn and early winter [46] and to some extent from biomass burning plumes from wildfires during summer/autumn. Another grouping (*referred to as Group B*) is proposed to include the amino acids Pro, Val, Ser, and Tyr (Tyr not discussed further due to too low accuracy of the applied analytical method) in being associated with terrestrial and marine aerosols. These amino acids exhibit a variety of reactivities in the atmosphere and may indicate a combination of various sources (plants, pollen, bacteria, phytoplankton) or an influence of both long-range and locally (within 1 day of transport) derived aerosols. Hydrophilic Ser was mostly not detected in the samples collected, consistent with it being highly reactive. Pro and Val are both hydrophobic, so the relation between the three compounds appears to be more affected by source differences than by similar chemical behaviors. Finally, a third group (*referred to as Group C*) is proposed to be those from coastal and marine phytoplankton and bacteria, namely Ile, Leu, and Thr. These are all relatively reactive amino acids and their instability indicates a local or medium distant source [18]. They are mainly released from Arctic Ocean surface water [47, 48].

### Correlation of Mapped Trajectories of Aerosols and Associated Amino Acid Profiles

In an attempt to elucidate source areas of the collected aerosol at the Zeppelin observatory, the calculated 5–10-day 3D backward trajectories were combined with the above-proposed groups of amino acids and their suggested sources. Note that the amino acids Asp and Ser were essentially not detected in this study and therefore will not be further discussed. As an additional parameter, we classified the vertical air movement of the trajectories into two groups referred to as the FT cluster and the BL cluster, respectively. In the FT cluster, the vertical component of the trajectory showed a subsiding pathway from the free troposphere (> 1000 m a.s.l.) during at least 75% of the last 5 days before arrival at the observatory. The BL cluster captured the samples for which the air spent 100% of the last 5 days below 500 m prior to the arrival at the receptor point.

**Figure 5:**
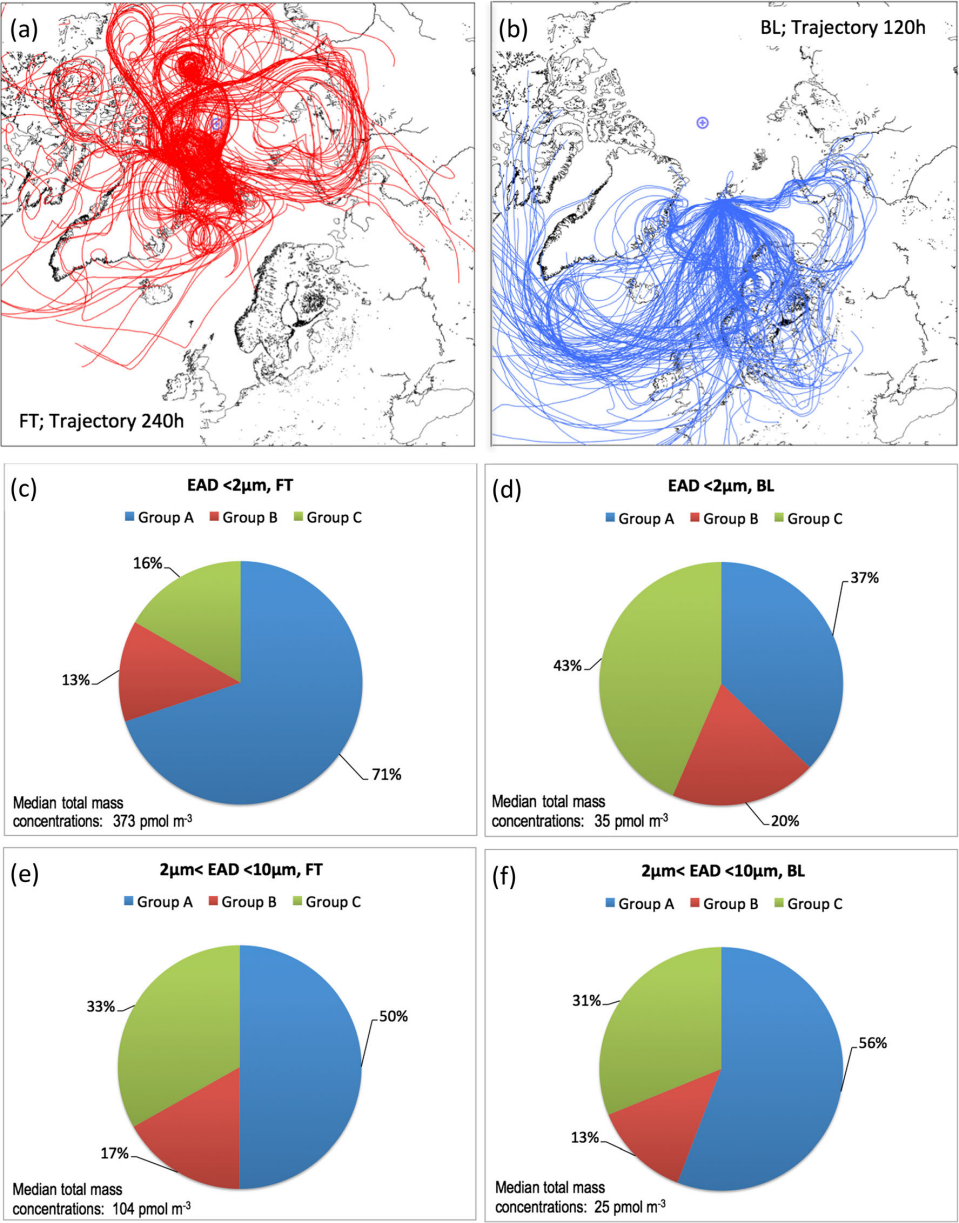
(**a**–**f**) The median relative abundance of TAAs in the fine (EAD < 2 μm) and coarse mode (2 < EAD < 10 μm) fractions within Groups A, B, and C for trajectory clusters FT and BL, respectively

The trajectory distributions of the FT and BL clusters, respectively, are graphed in Figure 5 together with the median relative abundance of amino acids in both the aerosol fine and coarse modes of *Groups A*, *B*, and *C*, respectively. With some overlap in the marginal ice zone and open waters reaching from the Greenland Sea to the Barents Sea, the geographic regions of the two trajectory subpopulations are largely complementary. The trajectories of the FT cluster (Figure 5a) came largely from Siberia (10 days ago) circumpolar over the pack ice and from the ice-covered archipelago north of Canada and Alaska merging from the direction of north Greenland towards the Greenland Sea—Fram Strait area. The average trajectory height of 1300 m a.s.l. during the last 5 days before trajectory arrival clearly points towards an FT origin of this cluster but for occasionally close contact with open sea during last 12–24 h before reaching the observatory. Figure 5b collects 5-day trajectories, with an average height within the marine BL that had passed over the open Norwegian and Greenland Seas from the northernmost parts of Scandinavia and from the non-ice covered Kara Sea area with some adjoining land contact.

The determined fine mode TAA fingerprints and the median total mass concentrations shown in Figure 5c, d reflect the distribution of the trajectories within the FT and BL clusters. The 10 times higher median total mass concentration (dominated by the mass of Gly in Group A) in the FT cluster (Figure 5c) relative to the BL cluster (Figure 5d) indicates long-distant aged aerosols possibly from residential heating. *Group C* amino acids (Leu being most important) predominate in the BL cluster (Figure 5d) indicating local or medium distant aerosols of coastal origin having distinct marine phytoplankton/microbial contributions. This was seemingly due to the low average trajectory travel height (below 500 m a.s.l.) that picked up TAA contribution from coastal and marine phytoplankton through the burst of film drops at sea-air interface. However, for more local emission of bacteria and their enzymes through jet drops (< 1 day of atmospheric residence time), the coarse mode aerosols would most likely have been gradually removed as the air passed over the Norwegian Sea. Moreover, within both trajectory groups, a less pronounced contribution at the 50th percentile level from Pro and Val (Figure 5c, d) in the fine mode was indicated from terrestrial sources.

**Figure 6:**
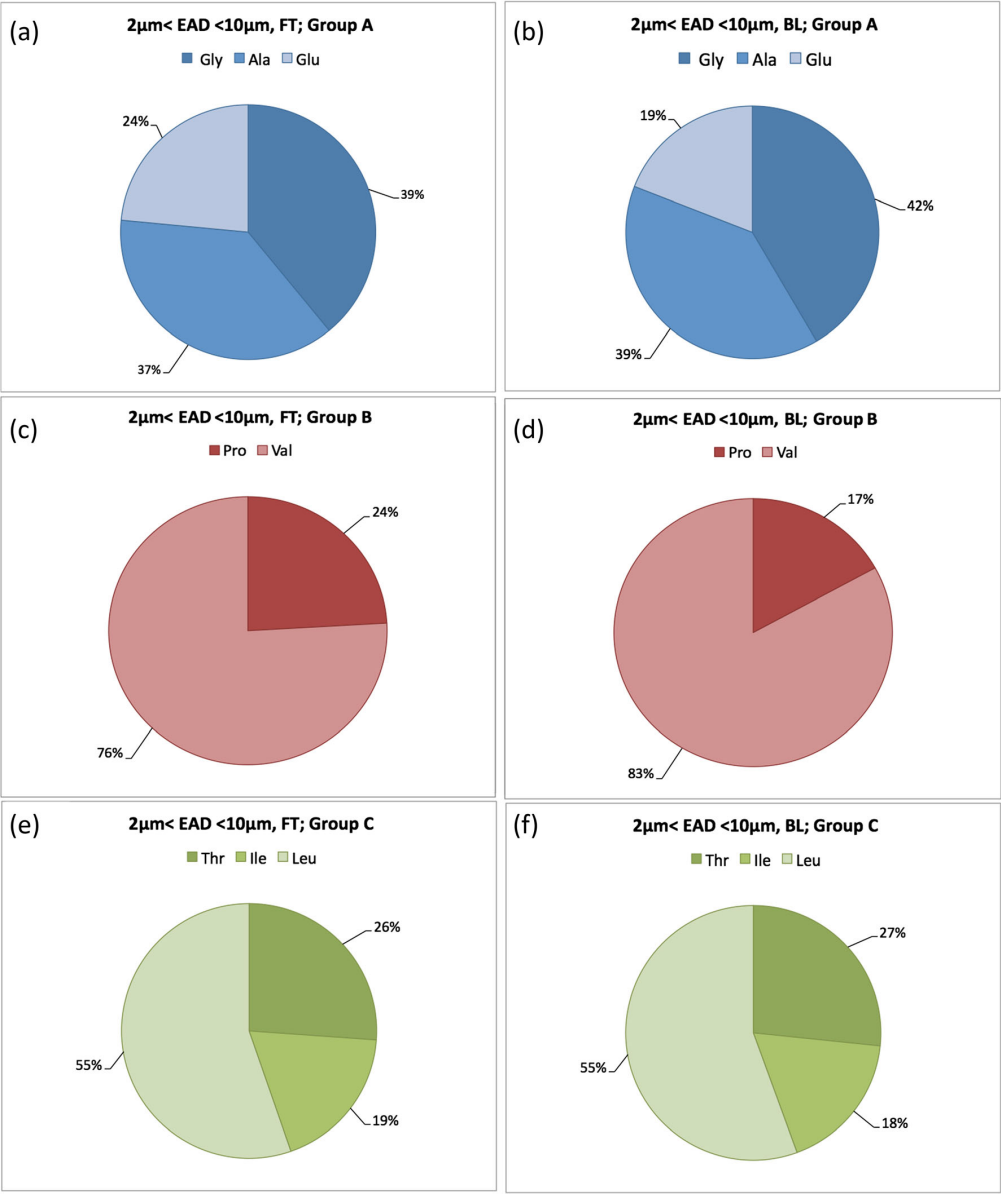
(**a**–**f**) The median relative abundance of TAAs in the coarse mode (2 < EAD < 10 μm) within Groups A, B, and C for each of the trajectory clusters, FT and BL

TAA fingerprints reflecting the coarse mode are shown in Figure 5e, f and represent a freshly (< 1 day atmospheric residence time) generated aerosol for which local marine sources are predominant. While the geographic distributions of the two trajectory clusters (Figure 5a, b) were largely complementary, the overlap in the marginal ice zone and open waters in the vicinity of Svalbard made the coarse mode (“jet drops”) fingerprints of Figure 5e, f to look very similar: i.e., at the 50th percentile level about 55%, 15 and 30% of TAA mass came from *Groups A*, *B*, and *C*, respectively. The similarity in TAA fingerprints between the trajectory clusters remains even if we explore in greater detail the coarse mode contributions of individual amino acids, for each of the *Groups* (*A*, *B*, and *C*). The results are plotted in terms of relative mass contribution at the 50th percentile level in Figure 6. Gly and Ala together provided close to 70% of the mass in *Group A* (Figure 6a, b), Pro contributed with about 80% of the *Group B* mass (Figure 6c, d), and in *Group C* (Figure 6e, f), the dominating amino acid was Thr accounting for 55% of the mass.

## Conclusions

An analytical methodology to investigate the content of total amino acids in atmospheric proteinaceous size-resolved aerosols is presented. Because our research focused on getting information about the total amount of proteinaceous material in the Arctic aerosols, free amino acids and polyamino acids were not separated in the present work. The present methodology, involving tagging with a fixed charge, gave low instrumental detection limits. An important achievement is that the tag induces specific MS fragmentation, thereby increasing the selectivity and improving both detectability and the certainty of identification. Furthermore, it allows essentially complete HPLC separation of 20 amino acid derivatives within only 11 min.

A noteworthy accomplishment in this study is the measurement of TAAs, as the sum of FAAs and PAAs, in size-resolved aerosols. An attempt was performed to elucidate source areas of the aerosols reaching the sampling station in the Arctic (Svalbard) covering a period in 2015 from September to December. Based on 3D back trajectories, the coarse mode as being short-lived in the atmosphere is indicative of local aerosol sources. The FT and BL cluster groups showed similar amino acid fingerprints despite covering different geographical regions. This is even true for very reactive amino acids, e.g., Thr, Ile, and Leu. This likely means that air parcels of both trajectory clusters picked up TAAs from local sources, i.e., Barents and Greenland Seas with contributions from marine phytoplankton and bacteria through bubble bursting at the sea-air interface. On the other hand, the fine particles with long residence time in the atmosphere yielded different amino acid signatures in the trajectory clusters, possibly reflecting the influence of either distant land (e.g., Siberia) or ocean (e.g., the North Sea) sources. The presence of large amounts of Gly in the fine aerosols is indicative of sources associated with residential heating. Similarly, Leu was also detected in the fine fraction at high relative concentrations and may have originated from sea surface phytoplanktons.

Much more research is needed regarding both levels and the roles of amino acids and proteins in cloud formation over the Arctic, as well the determination of the sources of the airborne aerosols. Importantly, the field of atmospheric chemistry studying transformations of amino acids and proteins and related effects on the formation of cloud condensation nuclei should gain from enhanced detection of biomolecules that are involved in cloud biogenesis.

## Electronic Supplementary Material

ESM 1 (DOCX 31 kb)
