# Supplementary material for: Measurements of Atmospheric Proteinaceous Aerosol in the Arctic Using a Selective UHPLC/ESI-MS/MS Strategy
Source: J Am Soc Mass Spectrom. 2018 Jul 17;30(1):161–73. doi: 10.1007/s13361-018-2009-8 (PMC6318243; doi:10.1007/s13361-018-2009-8)
Supplement: Supplementary file 1 — (DOCX 31 kb) [file 13361_2018_2009_MOESM1_ESM.docx]

**Electronic Supplementary Material**

**Table S-1.** The experimentally found amino acid composition (%) of human serum albumin obtained after using hydrolysis (TFA:HCl, 165ºC, 25 min), and the corresponding theoretical values.

| Human Serum Albumin amino acid composition (%) | | | |
| --- | --- | --- | --- |
| Amino acid | Experimental^1^ | Theoretical^4^ | Accuracy % |
| Lys | 10.2 ± 0.3 | 9.85 | 104 |
| Gly | 1.2 ± 0.1 | 2.13 | 56 |
| Ala | 10.2 ± 0.5 | 10.3 | 99 |
| Ser | 3.2 ± 0.2 | 4.60 | 70 |
| Pro | 5.0 ± 0.3 | 3.94 | 127 |
| Val | 6.1 ± 0.2 | 7.06 | 86 |
| Thr | 3.0 ± 0.1 | 4.76 | 63 |
| Cys^2^ | 3.0 ± 0.2 | 2.88^3^ | 104 |
| Ile | 1.7 ± 0.03 | 1.48 | 113 |
| Leu | 11.3 ± 1.1 | 10.5 | 108 |
| Asp+Asn^3^ | 4.4 ± 0.1 | 8.7 | 51 |
| Glu+Gln^3^ | 13.4 ± 0.2 | 13.5 | 99 |
| Met | 1.0 ± 0.1 | 1.15 | 87 |
| His | 2.3 ± 0.1 | 2.63 | 87 |
| Phe | 7.2 ± 0.2 | 5.75 | 125 |
| Arg | 3.4 ± 0.5 | 4.43 | 77 |
| Tyr | 9.2 ± 1.0 | 3.12 | 295 |
| Trp | 1.6 ± 0.1 | 0.33 | 484 |
|  |  |  |  |

^1^ N=3

^2^ Cys was detected as cystine in this study.

^3^Asn and Gln were completely converted to corresponding Asp and Glu at hydrolysis

^4^ <http://www.uniprot.org/uniprot/Q56G89>

**Table S-2.** MS operating parameters and selected MRM transitions for C_4_-NA-NHS

Derivatized amino acids. Cys was measured as cystine.

| Optimized parameters of ESI-MS/MS | | MRM settings | | | | |
| --- | --- | --- | --- | --- | --- | --- |
| Spray voltage (V) | 3000 |  | Transition | CE (eV) | Start (min) | Stop (min) |
| Source temperature (°C) | 150 | Lys | 235>106 | 25 | 6.5 | 7.5 |
| Desolvation temperature (°C) | 600 |  | 235>207 | 15 | 6.5 | 7.5 |
| Cone gas flow (L/h) | 300 | Gly | 237>106 | 30 | 1.5 | 2.3 |
| Desolvation gas flow (L/h) | 800 |  | 237>191 | 15 | 1.5 | 2.3 |
| Cone voltage (V) | 25 | Ala | 251>106 | 26 | 3.8 | 4.6 |
| Dwell time (ms) | 100 |  | 251>205 | 16 | 3.8 | 4.6 |
|  |  | Ser | 267>106 | 25 | 1 | 2 |
|  |  |  | 267>221 | 16 | 1 | 2 |
|  |  | Pro | 277>106 | 25 | 3.2 | 4.2 |
|  |  |  | 277>233 | 20 | 3.2 | 4.2 |
|  |  | Val | 279>106 | 25 | 6.2 | 7.5 |
|  |  |  | 279>233 | 16 | 6.2 | 7.5 |
|  |  | Thr | 281>106 | 30 | 3.7 | 5.9 |
|  |  |  | 281>235 | 18 | 3.7 | 5.9 |
|  |  | Cys | 282>106 | 35 | 7.3 | 8.3 |
|  |  |  | 282>161 | 15 | 7.3 | 8.3 |
|  |  | Leu | 293>106 | 30 | 7.7 | 9.2 |
|  |  |  | 293>247 | 20 | 7.7 | 9.2 |
|  |  | Ile | 293>106 | 30 | 7.7 | 9.2 |
|  |  |  | 293>247 | 20 | 7.7 | 9.2 |
|  |  | Asn | 294>106 | 35 | 0 | 1.4 |
|  |  |  | 294>235 | 18 | 0 | 1.4 |
|  |  | Asp | 295>106 | 30 | 1.5 | 2.6 |
|  |  |  | 295>205 | 20 | 1.5 | 2.6 |
|  |  | Gln | 308>106 | 27 | 2.1 | 3.2 |
|  |  |  | 308>291 | 18 | 2.1 | 3.2 |
|  |  | Glu | 309>106 | 30 | 3.4 | 4.3 |
|  |  |  | 309>263 | 20 | 3.4 | 4.3 |
|  |  | Met | 311>106 | 30 | 6.5 | 7.4 |
|  |  |  | 311>217 | 20 | 6.5 | 7.4 |
|  |  | His | 317>106 | 35 | 0 | 1.5 |
|  |  |  | 317>271 | 20 | 0 | 1.5 |
|  |  | Phe | 327>106 | 30 | 8.5 | 9.4 |
|  |  |  | 327>281 | 20 | 8.5 | 9.4 |
|  |  | Arg | 336>106 | 35 | 1.5 | 2.4 |
|  |  |  | 336>292 | 20 | 1.5 | 2.4 |
|  |  | Tyr | 343>106 | 30 | 5.9 | 6.8 |
|  |  |  | 343>297 | 20 | 5.9 | 6.8 |
|  |  | Try | 366>106 | 35 | 9 | 10 |
|  |  |  | 366>320 | 20 | 9 | 10 |
|  |  | Leu-D_3_ | 296>106 | 35 | 7.7 | 9.2 |
|  |  |  | 296>250 | 30 | 7.7 | 9.2 |
|  |  | Gln-D_5_ | 313>106 | 35 | 2.1 | 3.2 |
|  |  |  | 313>296 | 30 | 2.1 | 3.2 |
|  |  | Glu -D_5_ | 314>106 | 40 | 3.4 | 4.3 |
|  |  |  | 314>268 | 35 | 3.4 | 4.3 |
|  |  | Phe-D_5_ | 332>106 | 40 | 8.5 | 9.4 |
|  |  |  | 332>286 | 35 | 8.5 | 9.4 |

**Table S-3.** Performance of the analytical method for C_4_-NA-NHS derivatized amino acids. N=3.

| Analyte | Signal^1^ | RT (min)^2^ | LOD^3^ | MLOD^4^ | RSD% | Accuracy (%) | | |
| --- | --- | --- | --- | --- | --- | --- | --- | --- |
|  | % | ±RSD% | (pg) | (fmol/m^3^) | 1.5 µM | 1.5 µM | 6 µM | 8µM |
| His | 90.4 | 0.92 ± 2.18% | 3.97 | 0.22 | 4.7 | 101.5 | 115.2 | 100.0 |
| Asn^5^ | 97.4 | 1.11 ± 0.39% | 5.89 | - | 13.3 | 102.3 | 103.8 | 104.3 |
| Ser | 77.6 | 1.50 ± 0.67% | 1.30 | 0.09 | 2.6 | 88.0 | 93.6 | 83.8 |
| Gly | 69.0 | 1.80 ± 0.61% | 0.86 | 0.06 | 4.1 | 94.8 | 107.9 | 107.3 |
| Arg | 70.1 | 1.94 ± 1.50% | 36.92 | 1.93 | 0.9 | 87.4 | 110.5 | 99.2 |
| Asp | 84.6 | 2.04 ± 0.49% | 4.02 | 0.24 | 6.7 | 105.0 | 107.7 | 95.5 |
| Gln^5^ | 72.7 | 2.59 ± 0.81% | 2.14 | - | 25.4 | 86.2 | 92.7 | 90.6 |
| Pro | 78.3 | 3.69 ± 0.48% | 2.58 | 0.16 | 0.5 | 93.3 | 101.2 | 105.4 |
| Glu | 94.1 | 3.85 ± 0.49% | 0.49 | 0.03 | 8.2 | 108.2 | 107.5 | 104.2 |
| Thr | 84.0 | 4.14 ± 0.34% | 1.25 | 0.08 | 3.9 | 98.7 | 97.5 | 97.7 |
| Ala | 72.9 | 4.17 ± 0.35% | 0.21 | 0.01 | 3.2 | 101.0 | 105.5 | 107.3 |
| Tyr^5^ | 77.3 | 6.33 ± 1.95% | 0.59 | - | 4.0 | 101.2 | 96.9 | 106.2 |
| Val | 79.0 | 6.65 ± 0.27% | 0.39 | 0.02 | 1.0 | 96.6 | 94.0 | 94.5 |
| Met | 64.6 | 6.82 ± 0.17% | 0.29 | 0.02 | 1.1 | 88.5 | 96.1 | 97.3 |
| Lys | 62.5 | 7.02 ± 0.42% | 1.78 | 0.07 | 2.5 | 84.9 | 97.5 | 97.4 |
| Cys^6^ | 84.9 | 7.7 ± 0.12% | 8.06 | 0.50 | 1.6 | 88.0 | 91.1 | 94.4 |
| Ile | 77.9 | 8.23 ± 0.12% | 0.36 | 0.02 | 1.1 | 103.7 | 90.9 | 95.7 |
| Leu | 73.8 | 8.50 ± 0.23% | 0.29 | 0.02 | 0.7 | 99.0 | 97.6 | 98.9 |
| Phe | 74.2 | 8.82 ± 0.14% | 0.46 | 0.02 | 1.2 | 103.8 | 101.3 | 100.1 |
| Trp^5^ | 90.5 | 9.29 ± 0.10% | 3.33 | - | 1.7 | 98.2 | 98.9 | 97.4 |
| ^1^ Signal % = 100 x slope matrix-adapted /Slope matrix-free limits of quantification (LOQ).  ^2^ RT=HPLC retention time  ^3^ Instrumental LOD as amount injected on column  ^4^ MLOD= method LOD based on the average sampling period of 60 h corresponding to a total air sampling volume of 57 m^3^.  ^5^ Included in the UHPLC/ESI-MS/MS method development, but not measured in the aerosols  ^6^ Cys was detected as cystine. | | | | | | | | |
